# Diagnostic pitfalls in a young adult with new diabetes

**DOI:** 10.1530/EDM-23-0024

**Published:** 2023-10-12

**Authors:** Natalie Below, Deborah Morrison, Ruth McGowan, Gregory C Jones

**Affiliations:** 1 Diabetes Centre, Gartnavel General Hospital, Glasgow, UK; 2 University of Glasgow, Glasgow, UK; 3 West of Scotland Centre for Genomic Medicine, Glasgow, UK

**Keywords:** Adolescent/young adult, Male, Asian - other, United Kingdom, Pancreas, Diabetes, genetics, Error in diagnosis/pitfalls and caveats, October, 2023

## Abstract

**Summary:**

A 20-year-old South Asian male presented with polyuria, polydipsia, HbA1c 81 mmol/mol, BMI 28.8 and family history of both type 1 and type 2 diabetes mellitus. As autoantibody testing was negative and c-peptide level demonstrated significant endogenous insulin secretion, type 1 diabetes was excluded. Given his age and family history, the differential diagnosis included maturity-onset diabetes of the young (MODY), a rare form of diabetes caused by a single-gene variant. A high probability of MODY was calculated and he was subsequently referred for genetic testing. Although a useful tool, the pre-test probability calculator for MODY is only validated in White Europeans. A heterogenous variant of unknown clinical significance of the NEUROD1 gene was detected, leading to gliclazide use with poor response. The patient responded well to metformin. Type 2 diabetes was considered the most likely diagnosis. This case highlights the diagnostic challenges in young patients of Asian ethnicity and the importance of interpreting genetic results of unknown significance within the clinical context. Ethnicity-specific BMI thresholds should be used when classifying patients as overweight or obese.

**Learning points:**

- Variants of unknown significance detected by genetic sequencing should be interpreted within the context of the patient’s other clinical parameters.
- It is important to use ethnicity-specific BMI thresholds for obesity.
- Diagnosis of type 2 diabetes mellitus at younger ages is becoming increasingly common.
- The pre-test probability calculator for MODY is only validated in White Europeans; although a useful guide, results should be interpreted with caution in patients of other ethnicities.

## Background

Maturity-onset diabetes of the young (MODY) is a form of diabetes caused by a single gene variant (1). MODY is genetically heterogenous, with alterations in at least 14 different genes reported, including neurogenic differentiation 1 (NEUROD1), a transcription factor involved in pancreatic beta cell maturation and maintenance. Variants in this gene have been demonstrated to cause beta-cell dysfunction, resulting in childhood- or adult-onset diabetes where the variants are heterozygous (1). This form of MODY is associated with obesity and, less commonly, intellectual disabilities with brain abnormalities. As MODY6 shows incomplete penetrance of diabetes, diet and oral hypoglycaemic agents tend to be effective management and insulin therapy is often not required (2). Monogenic diabetes typically develops before the age of 25, runs in families and has autosomal dominant inheritance. It is often misdiagnosed as either type 1 or type 2 diabetes (3). An accurate diagnosis is important, as diabetes management differs depending on the aetiology/type (1).

Type 2 diabetes mellitus (T2DM) is known to be associated with the following risk factors: age, obesity, ethnicity, and family history. T2DM is traditionally thought to develop later in life than other types of diabetes, with a peak incidence at around 55 years of age (4). However, with an increase in childhood and adolescent obesity, diagnosis of T2DM at younger ages is becoming increasingly common (5).

## Case presentation

A 20-year-old male of Southeast Asian ethnicity presented with polyuria and polydipsia. At diagnosis, random glucose was 10.1 mmol/L, HbA1c was 81 mmol/mol, weight was 93 kg and BMI was 28.8, and there was no ketonuria. His father was reported to have type 1 diabetes diagnosed aged 20 years, and his mother with type 2 diabetes at 40 years of age. The patient was initially treated with metformin and lifestyle advice and was referred to a specialist diabetes clinic due to his age <40 years as per local guidelines.

## Investigation

**Figure 1:**
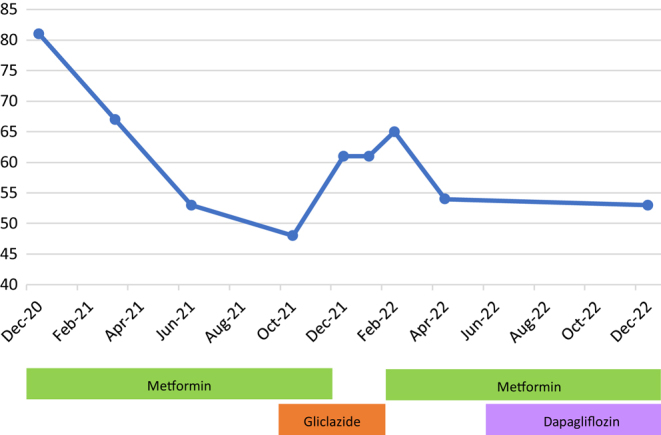
Timeline of HbA1c results. Graph showing the effect of different antidiabetic medications on the patient’s HbA1c over time.

Three months after diagnosis, his c-peptide was found to be 996 pmol/L, suggesting significant endogenous insulin, and GAD/IA2 antibodies were negative. With metformin alone, his HbA1c improved to 67 mmol/mol. Type 1 diabetes was therefore excluded. By 6 months, a further improvement in HbA1c was demonstrated by FreeStyle Libre flash glucose monitoring (Abbott Diabetes Care) to an estimate of 53 mmol/mol on metformin alone, as depicted in Fig. 1.

The Exeter MODY probability calculator estimated a 58% probability of MODY. Next-generation sequencing was subsequently undertaken for a 34-gene MODY panel by the East of Scotland Regional Genetics Laboratory. A heterogenous gene variant c.[890A>G];[=] p.(Tyr297Cys) was detected in the neuronal differentiation 1 (NEUROD1) gene and, in accordance with the Association for Clinical Genomic Science (ACGS) guidance, reported a variant of unknown clinical significance.

The result raised suspicion of a rare form of MODY caused by variants in the NEUROD1 gene; however, the patient did not have clinical features of this form of MODY, which can be associated with neurological abnormalities such as sensory–neural deafness, visual impairment and developmental delay (6). Brain MRI (performed on the advice from clinical genetics) was normal.

A referral was made to clinical genetics and co-segregation of the variant with three other affected family members was advised to confirm pathogenicity. This was unfortunately not possible as the patient’s family lived outside the UK.

## Treatment

Given the clinical suspicion of MODY, decreased insulin secretion was suspected; therefore, at 7 months post diagnosis, gliclazide 80 mg bid was commenced. This resulted in a slight improvement in glycaemic control, with an estimated HbA1c from flash glucose monitoring of 48 mmol/mol. Two months later, metformin was reduced and then stopped. Following this, a noticeable worsening of glycaemic control (Libre glucose management indicator HbA1c 61 mmol/mol) was observed. Gliclazide was stopped and metformin restarted, following which glycaemic control improved again. After an increase in metformin dose to 1 g bid, the patient’s HbA1c further improved to an estimated 54 mmol/mol. A timeline of these effects of different medications on the patient’s HbA1c levels is illustrated by Fig. 1 .

## Outcome and follow-up

The patient was diagnosed with type 2 diabetes mellitus and is followed up routinely in the diabetes outpatient clinic. He is currently treated with metformin and dapagliflozin.

## Discussion

Given this patient’s age of <25 years, presence of diabetes in two consecutive family generations, absence of β-cell autoantibodies, and preserved endogenous insulin secretion with a serum C-peptide level of >200 pmol/L, he met the criteria for consideration of a diagnosis of MODY (1).

NEUROD1 (MODY 6) is very rare. Whilst it can present with obesity and intellectual disability, diabetic ketoacidosis or ketosis may be the only abnormality and age at onset of diabetes is variable (2). Genetic testing is essential for diagnosis; however, routine genetic testing is expensive and selection for testing based on pre-test probability is preferable. The MODY probability calculator was developed in 2012 to address this (7). Although this tool shows good discrimination between monogenic and type 1 and type 2 diabetes, it is only validated in White Europeans (8). Therefore, the probability of MODY predicted by the calculator in this case is potentially inaccurate.

The NEUROD1 variant detected in this patient had not been reported in literature either as a pathogenic variant in affected individuals or general population (absent from ClinVar and gnomAD databases) (9, 10). Based on the current evidence and guidelines for variant reporting, this was classified as a variant of unknown significance. Checking for segregation of a gene variant by offering testing to affected and unaffected family members can help with the classification of uncertain variants; however, this was not possible in this family. Furthermore, the family history would have made this challenging as both parents had diabetes. The patient reported that his father and mother have type 1 and type 2 diabetes respectively, but we were unable to confirm this. Given the parents’ age and ethnicity profiles, differentiating between type 1 diabetes, type 2 diabetes and MODY would have been challenging without conducting further investigations.

According to ACGS classifications guidelines, response to sulphonylurea can be used as phenotypic evidence at a supporting level in other types of MODY (HNF1A/4A) (11). Although the patient had an initial improvement in glycaemic control following addition of gliclazide, he was clearly more responsive to metformin, and sulfonylurea responsiveness is not known to be a feature of NEUROD1. A characteristic of MODY 6 which can contribute to misdirection when considering diagnosis in young adults is that it can present with initial ketosis which can suggest type 1 diabetes. For reasons unknown and despite this those with MODY 6 can usually be treated with oral therapy.

Not only is ethnicity an independent risk factor for developing T2DM; it also affects the way in which BMI should be interpreted. A population-based cohort study in England suggests that a lower BMI cut-off of 23.9kg/m^2^ should be used to define obesity based on the risk of developing T2DM in South Asian adults, as this was found to be equivalent to the BMI cut-off for obesity of 30.0 kg/m^2^ set for white populations when examining age-adjusted and sex-adjusted incidence (12). This is of important consideration when presented with a case such as the patient in this report, whose BMI was <30 and only classifies as ‘obese’ when ethnicity is taken into account (13). Given his South Asian background, his BMI of 28.8 kg/m^2^ in fact correlates to a BMI >30.0 kg/m^2^ in white populations.

The diagnosis of T2DM at younger ages is becoming increasingly common (5) and can be challenging, as demonstrated in the case presented, where the patient’s age of 20 years was considered to be more in keeping with a potential diagnosis of MODY.

The case represents the challenge in diagnosing diabetes in young individuals of Asian ethnicity and in differentiating T2DM from MODY. It also highlights the challenges of interpreting the results of genetic sequencing, which may not provide definitive answers.

## Declaration of interest

There is no conflict of interest that could be perceived as prejudicing the impartiality of the research reported.

## Funding

This research did not receive any specific grant from any funding agency in the public, commercial or not-for-profit sector.

## Patient consent

Written informed consent for publication of their clinical details was obtained from the patient.

## Author contribution statement

NB wrote the manuscript. GCJ, DM, and RM reviewed and edited the manuscript and were responsible for the clinical management of the case.
